# Relationship between DNA Methylation Profiles and Active Tuberculosis Development from Latent Infection: a Pilot Study in Nested Case-Control Design

**DOI:** 10.1128/spectrum.00586-22

**Published:** 2022-04-21

**Authors:** Ying Du, Xu Gao, Jiaoxia Yan, Haoran Zhang, Xuefang Cao, Boxuan Feng, Yijun He, Yongpeng He, Tonglei Guo, Henan Xin, Lei Gao

**Affiliations:** a NHC Key Laboratory of Systems Biology of Pathogens, Institute of Pathogen Biology, and Center for Tuberculosis Research, Chinese Academy of Medical Sciences and Peking Union Medical College, Beijing, People’s Republic of China; b Department of Occupational and Environmental Health Sciences, School of Public Health, Peking University, Beijing, People’s Republic of China; c Center for Diseases Control and Prevention of Zhongmu, Zhengzhou, People’s Republic of China; Shandong First Medical University

**Keywords:** tuberculosis, latent tuberculosis infection, DNA methylation, biomarker, nested case-control study, tuberculosis

## Abstract

Individuals with latent tuberculosis infection (LTBI) were regarded as an enormous reservoir of cases with active tuberculosis (TB). To strengthen LTBI management, biomarkers and tools are urgently required for identifying and ruling out active TB in a fast and effective way. Based on an open-label randomized controlled trial aiming to explore short-course LTBI treatment regimens, DNA methylation profiles were retrospectively detected to explore potential biomarkers, which could discriminate active TB from LTBI. The Infinium MethylationEPIC BeadChip array was used to analyze genomewide DNA methylation levels for 15 persons with LTBI who later developed active TB and for 15 LTBI controls who stayed healthy. The differentially methylated CpGs (dmCpGs) located in the promoter regions pre- and post-TB diagnosis were selected (*P* < 0.05 and |Δβ|>0.10) and evaluated by receiver operating characteristic (ROC) analysis. Eight dmCpGs were identified to be associated with TB occurrence; six were located in hypermethylated genes (cg02493602, cg02206980, cg02214623, cg12159502, cg14593639, and cg25764570), and two were located in hypomethylated genes (cg02781074 and cg12321798). ROC analysis indicated that the area under curve (AUC) of these eight dmCpGs ranged from 0.72 to 0.84. Given 90% sensitivity, the specificity was highest for cg14593639 at 66.67%. The combination analysis indicated that “cg02206980 + cg02214623 + cg12159502 + cg12321798” showed the best performance, with an AUC of 0.88 (95% confidence interval [CI]: 0.72, 0.97), a sensitivity of 93.33% (95% CI: 70.18%, 99.66%), and a specificity of 86.67% (95% CI: 62.12%, 97.63%). Our preliminary results indicate the potential value of the DNA methylation level as a diagnostic biomarker for discriminating active disease in LTBI testing. This finding requires further verification in independent populations with large sample sizes.

**IMPORTANCE** Approximately a quarter of the world population had been infected with Mycobacterium tuberculosis, and about 5 to 10% of these individuals might develop active disease in their lifetimes. As a critical component of the “end TB strategies,” preventive treatment was shown to protect 60 to 90% of high-risk LTBIs from developing active disease. Developing new TB screening tools based on blood-based biomarkers, which could identify and rule out active TB from LTBI, are prerequisite before initialing intervention. We tried to explore potential DNA methylation diagnostic biomarkers through retrospectively detected DNA methylation profiles pre- and post-TB diagnosis. Eight dmCpGs were identified, and the combination of “cg02206980 + cg02214623 + cg12159502 + cg12321798” showed a sensitivity of 93.33% and a specificity of 86.67%. The preliminary results provided new insight into detecting the DNA methylation level as a potential tool to distinguish TB from LTBI.

## INTRODUCTION

Globally, tens of millions of people were reported to be infected with Mycobacterium tuberculosis, and approximately 5 to 10% of them might develop active disease in their lifetimes (1). Individuals with latent tuberculosis infection (LTBI) were regarded as an enormous reservoir of active tuberculosis (TB) cases, the procession of which is usually complex and dynamic (2). Comprehensive strategies had been implemented to control such seedbeds of TB. Among them, preventive treatment could effectively reduce the risk of active TB development with an efficacy of 60 to 90% (3). However, how to identify target populations with the high priority for preventive treatment was a big challenge for LTBI management, especially in resource-limited countries. Identifying and ruling out active TB from LTBI with advanced diagnostics tools were prerequisite before starting preventive treatment. The World Health Organization (WHO) recommends that new diagnostic tests for TB should be low cost, easy to use, and noninvasive and achieve high sensitivity and specificity (4). Thus, blood-based, host-derived immune response biomarkers, which could reflect a broad view of the host response to TB provided a promising insight and are urgently required for identifying TB at an early stage among at-risk populations with LTBI (5–9).

Recent studies suggested that M. tuberculosis could alter the host epigenome to modulate the transcriptional machinery by either activation or the suppression of key immune genes involved in immune response or pathogen persistence (10). Since epigenetics can bridge the gaps between the host, M. tuberculosis, and the environment, it might have great potential in predicting TB development. DNA methylation is the most widely studied epigenetic marker, and cytosine-guanine dinucleotide (CpG) methylation is central to many biological processes and human diseases (11–14). Previous studies reported that varied DNA methylation might be associated with TB risk (15, 16). However, most of these studies used case-control or cross-sectional study designs which could not allow for a temporal analysis of the relationship between DNA methylation and TB occurrence. In addition, few reported biomarkers could achieve the 90% sensitivity and 70% specificity set by WHO target product profile (TPP) benchmarks for screening TB. In our previous randomized controlled trial aiming to explore short-course LTBI treatment regimens, individuals with treated and untreated LTBI were monitored for 5 years to track the development of active TB. Based on this study, the current pilot study retrospectively detected DNA methylation profiles among individuals who developed active TB from LTBI in order to explore potential markers, which could be used to identify and rule out active disease.

## RESULTS

### Characteristics of the study participants.

**FIG 1:**
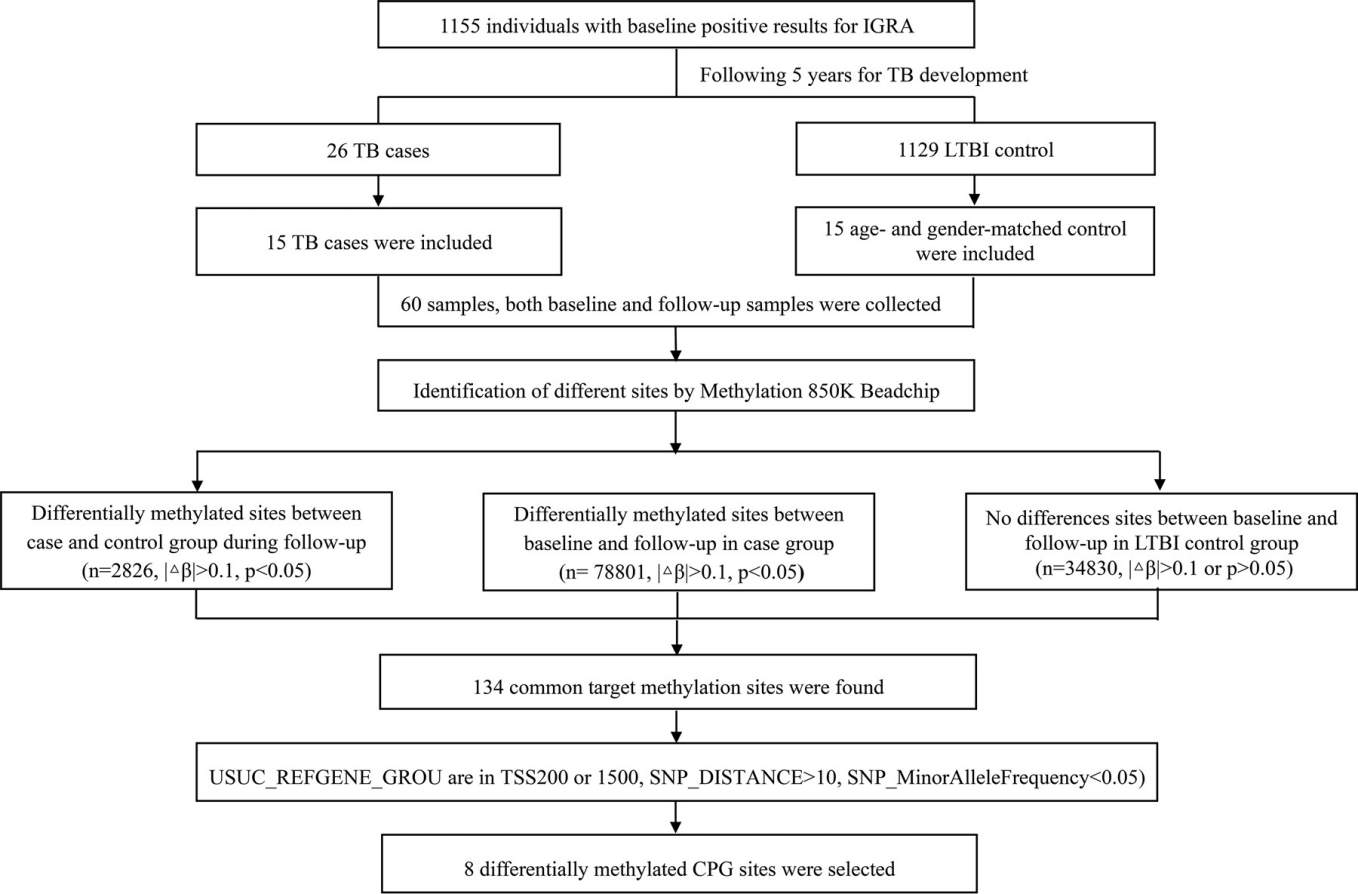
Flow chart of the study. By 2020, 26 TB incidence cases were identified during the 5-year follow-up period among 1,155 untreated individuals with LTBI. Fifteen TB cases and fifteen age- and gender-matched LTBI controls were included in the study. Two sets of blood samples for each subject were collected, including one sample at baseline and one sample at diagnosis for those developed active TB or at terminal survey for those stayed healthy during follow-up. The samples at different time points were detected by EPIC BeadChip array to estimate the genomewide DNA methylation patterns. Differentially methylated CpG loci between the case group and the control group were detected, including 2,826 dmCpGs at follow-up. A total of 78,110 dmCpGs changed significantly pre- and post-TB occurrence. Among these, no change was observed throughout the study in the control group for 134 CpG sites; eight of them in the promoter regions (six hypermethylated genes and two hypomethylated genes) were regarded as candidate CpGs for further analysis. Promoters were defined as regions located between 1,500 bp upstream of TSS and 200 bp downstream of TSS and genes containing multiple differentially methylated probes. IGRA, interferon gamma release assays; LTBI, latent tuberculosis infection; PBMC, peripheral blood mononuclear cells; TB, tuberculosis; TSS, transcriptional start sites.

As shown in Fig. 1, 15 TB cases developed from LTBI, and 15 age- and gender-matched LTBI controls stayed healthy were included in the present study. The detail diagnostic information of the TB cases was presented in Table S1 in the supplemental material. Two samples at different time points were detected for each participant by EPIC BeadChip array to estimate the genomewide DNA methylation patterns.

Table 1 presents the major baseline characteristics of the 30 study participants. The median age was 65 years (range, 62 to 68 years), the majority being male (73.33%, 22/30). No significant difference was found between the two groups with respect to baseline interferon gamma release assay results, smoking history, and alcohol drinking status. Those who developed TB were found to have a lower body mass index than those who remained free of TB (*P = *0.025).

**TABLE 1:** Characteristics of the study participants with LTBI^*a*^

Parameter	Participants who developed active TB during follow-up (*n* = 15)	Participants who stayed healthy during follow-up (*n* = 15)	*P* ^*b*^
Median age, yr (Q25–Q75)	67.00 (61.00–68.00)	65.00 (62.00,67.00)	0.437†
Gender, *n* (%)			
Male	11 (73.33)	11 (73.33)	1.000#
Female	4 (26.67)	4 (26.67)	
Median BMI, kg/m^2^ (Q25–Q75)	21.99 (20.99–22.72)	24.03 (22.00–28.08)	0.025†
Ever smoked, *n* (%)			
Yes	8 (53.33)	9 (60.00)	1.000#
No	7 (46.67)	6 (40.00)	
Current alcohol drinking, *n* (%)			
Yes	7 (46.67)	6 (40.00)	1.000#
No	8 (53.33)	9 (60.00)	
Median IFN-γ releasing level at baseline IGRA testing, IU/mL (Q25–Q75)	1.44 (0.84–3.33)	2.12 (1.55–3.59)	0.151†

a Q25–Q75, 25th to 75th percentiles; LTBI, latent tuberculosis infection; TB, tuberculosis; BMI, body mass index; IFN-γ, interferon gamma; IGRA, interferon gamma release assays.

b †, Wilcoxon rank sum test; #, Fisher exact test.

### Differentially expressed DNA methylation patterns between various groups.

**FIG 2:**
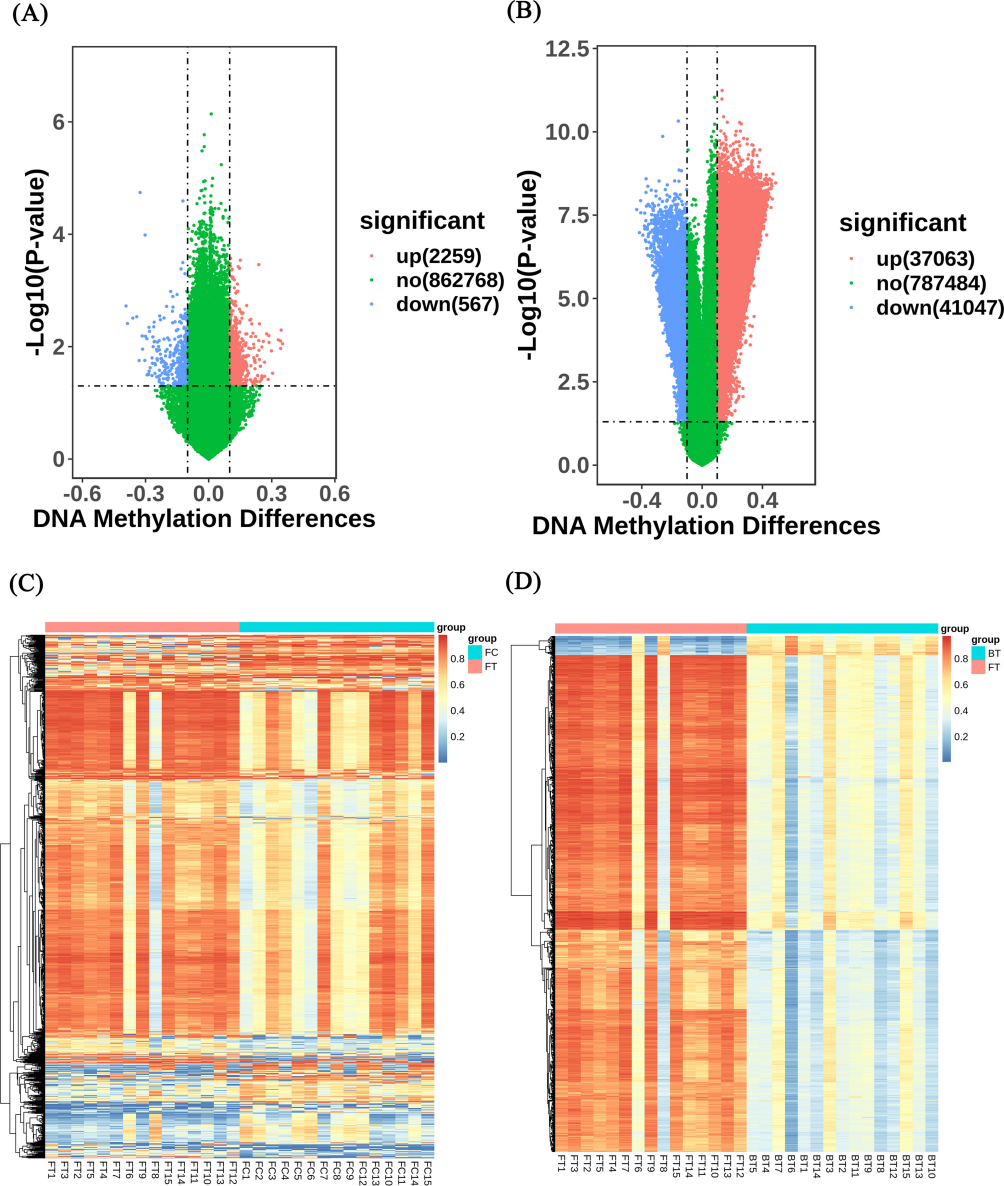
Visualization of differentially methylated probes. (A) Volcano plot of differentially methylated CpG sites between the case group and the control group at follow-up. (B) Volcano plot of differentially methylated CpG sites between baseline and follow-up in case group. The *x* axis represents the magnitude of the difference in signal intensity between the groups for each probe in the microarray, expressed as Δβ = β (group 1) − β (group 2). The *y* axis represents the −log_10_ (*P* value), with a *P* value of 0.05. Significantly different sites (*P < *0.05 and |Δβ|> 0.10) are highlighted in red and blue. (C) Hierarchical clustering of the variable CpG sites derived from the case group and the control group at follow-up. (D) Hierarchical clustering of the variable CpG sites derived from the baseline and the follow-up in the case group. Different groups are represented: FC is the control group during follow-up, FT is the TB case group during follow-up. BT is the TB case group at baseline. Methylation levels are expressed as β values from 0 (blue, completely unmethylated) to 1 (red, fully methylated).

To acquire candidate methylated CpG sites meeting preset criteria, the methylated statuses of 863,159 CpG sites in 60 blood samples were analyzed using the EPIC BeadChip array. A total of 2,826 dmCpGs discriminately expressed between case group and control group at follow-up (Fig. 2A and C) and 78,110 dmCpGs changed significantly pre- and post-TB occurrence (Fig. 2B and D). Among them, no significant change was observed throughout the study in control group for 134 methylated CpG sites. Finally, eight of them in the promoter regions were regarded as candidate CpG sites for further analysis; six were located in hypermethylated genes (cg02493602, cg02206980, cg02214623, cg12159502, cg14593639, and cg25764570), and two were located in hypomethylated genes (cg02781074 and cg12321798). The basic information of these eight dmCpGs is shown in Table 2.

**TABLE 2:** Basic information of the identified differentially methylated CpG sites^*a*^

Target_ID	Refseq gene	CpG island region	β				FT vs FC		FT vs BT		FC vs BC	
			FT	FC	BT	BC	Δβ	*P*	Δβ	*P*	Δβ	*P*
cg02493602	ME3	S_Shore	0.4645	0.3269	0.2812	0.2791	0.1376	0.015	0.1833	0.002	0.0478	0.279
cg02206980	SIRT5	N_Shore	0.4923	0.3572	0.2843	0.2629	0.1351	0.019	0.2080	<0.001	0.0943	0.018
cg02214623	GNB2L1	N_Shore	0.5380	0.4229	0.3598	0.3428	0.1151	0.001	0.1782	<0.001	0.0801	0.003
cg12159502	SIRT1	N_Shore	0.6655	0.5574	0.5612	0.4998	0.1081	0.002	0.1043	<0.001	0.0576	0.079
cg14593639	ADGRG6	N_Shore	0.6434	0.5395	0.5164	0.4584	0.1039	0.011	0.1270	0.001	0.0811	0.020
cg25764570	HLA-DRA	NA	0.6900	0.5884	0.5718	0.5213	0.1016	0.004	0.1181	0.008	0.0670	0.090
cg02781074	GGACT	Island	0.4971	0.6038	0.6225	0.6705	–0.1067	0.015	–0.1254	0.009	–0.0667	0.071
cg12321798	FLJ44635	NA	0.5176	0.6178	0.7018	0.6936	–0.1002	0.028	–0.1842	<0.001	–0.0758	0.060

a Δβ = mean β value (group 1) – mean β value (group 2). FT, tuberculosis case group during follow-up; FC, control group during follow-up; BT, tuberculosis case group at baseline; BC, control group at baseline; NA, not applicable.

### Diagnostic value of TB-associated differentially methylated CpG sites.

The diagnostic values of the eight dmCpGs were identified using ROC curves. The results indicated that the areas under the ROC curve (AUC) of these eight dmCpGs ranged from 0.72 to 0.84. Among them, cg12159502 located in Sirt1 presented the best AUC of 0.84 (95% confidence interval [CI] = 0.66 to 0.95) with a sensitivity of 73.33% (95% CI = 48.05 to 89.10%) and a specificity of 86.67% (95% CI = 62.12 to 97.63%). Given 90% sensitivity, the specificities were highest for cg14593639 of 66.67% (Table 3). We then calculated the performance of different combinations for these eight dmCpGs. Among 247 different combinations (28 combinations for any two CpGs, 56 combinations for any three CpGs, 70 combinations for any four CpGs, 56 combinations for any five CpGs, 28 combinations for any six CpGs, 8 combinations for any seven CpGs, and 1 combination for all), 52 combinations met the WHO TPP benchmarks (see Table S2 in the supplemental material). Table 4 presents the combinations with the best performance for each category. Among them, the combination of “cg02206980 + cg02214623 + cg12159502 + cg12321798” showed the best performance, with a sensitivity of 93.33% and a specificity of 86.67%.

**TABLE 3:** Performance of eight identified methylated CpG sites in discriminating active TB from LTBI^*a*^

Target_ID	Refseq gene	AUC (95% CI)	*P*	% sensitivity and specificity (95% CI)			
				Maximum Youden index		WHO TPP benchmark	
				Sensitivity	Specificity	Sensitivity	Specificity
cg02493602	ME3	0.76 (0.58–0.94)	0.005	80.00 (54.81–92.95)	73.33 (48.05–89.10)	93.33 (70.18–99.66)	13.33 (2.37–37.88)
cg02206980	SIRT5	0.76 (0.56–0.96)	0.011	80.00 (54.81–92.95)	80.00 (54.81–92.95)	93.33 (70.18–99.66)	0.00 (0.00–20.39)
cg02214623	GNB2L1	0.84 (0.69–0.98)	<0.001	86.67 (62.12–97.63)	66.67 (41.71–84.82)	93.33 (70.18–99.66)	53.33 (30.12–75.19)
cg12159502	SIRT1	0.84 (0.69–0.99)	<0.001	73.33 (48.05–89.10)	86.67 (62.12–97.63)	93.33 (70.18–99.66)	53.33 (30.12–75.19)
cg14593639	ADGRG6	0.76 (0.57–0.95)	0.008	93.33 (70.18–99.66)	66.67 (41.71–84.82)	93.33 (70.18–99.66)	66.67 (41.71–84.82)
cg25764570	HLA-DRA	0.80 (0.63–0.96)	0.001	86.67 (62.12–97.63)	60.00 (35.75–80.18)	93.33 (70.18–99.66)	40.00 (19.82–64.25)
cg02781074	GGACT	0.76 (0.58–0.93)	0.004	93.33 (70.18–99.66)	53.33 (30.12–75.19)	93.33 (70.18–99.66)	53.33 (30.12–75.19)
cg12321798	FLJ44635	0.72 (0.53–0.91)	0.029	53.33 (30.12–75.19)	93.33 (70.18–99.66)	93.33 (70.18–99.66)	33.33 (0.00–20.39)

a TB, tuberculosis; LTBI, latent tuberculosis infection; AUC, area under the receiver operator characteristic curve; CI, confidence interval; WHO TPP, World Health Organization target product profile.

**TABLE 4:** Performance of different combinations of the 8 identified methylated CpG sites in discriminating active TB from LTBI^*a*^

Combination	AUC (95% CI)	*P*	% sensitivity and specificity (95% CI)			
			Maximum Youden index		WHO TPP benchmarks	
			Sensitivity	Specificity	Sensitivity	Specificity
cg02206980 + cg02214623 + cg12159502	0.89 (0.72–0.97)	<0.001	93.33 (70.18–99.66)	80.00 (54.81–92.95)	93.33 (70.18–99.66)	80.00 (54.81–92.95)
cg02206980 + cg02214623 + cg12159502 + cg12321798	0.88 (0.72–0.97)	<0.001	93.33 (70.18–99.66)	86.67 (62.12–97.63)	93.33 (70.18–99.66)	86.67 (62.12–97.63)
cg02206980 + cg02214623 + cg12159502 + cg14593639 + cg12321798	0.89 (0.72–0.97)	<0.001	93.33 (70.18–99.66)	86.67 (62.12–97.63)	93.33 (70.18–99.66)	86.67 (62.12–97.63)
cg02206980 + cg02214623 + cg12159502 + cg25764570 + cg12321798	0.89 (0.72–0.97)	<0.001	93.33 (70.18–99.66)	86.67 (62.12–97.63)	93.33 (70.18–99.66)	86.67 (62.12–97.63)
cg02206980 + cg02214623 + cg12159502 + cg14593639 + cg25764570 + cg12321798	0.89 (0.72–0.97)	<0.001	93.33 (70.18–99.66)	86.67 (62.12–97.63)	93.33 (70.18–99.66)	86.67 (62.12–97.63)
cg02206980 + cg02214623 + cg12159502 + cg14593639 + cg25764570 + cg02781074 + cg12321798	0.90 (0.73–0.98)	<0.001	93.33 (70.18–99.66)	80.00 (54.81–92.95)	93.33 (70.18–99.66)	80.00 (54.81–92.95)
cg02493602 + cg02206980 + cg02214623 + cg12159502 + cg14593639 + cg25764570 + cg02781074 + cg12321798	0.90 (0.73–0.98)	<0.001	86.67 (62.12–97.63)	86.67 (62.12–97.63)	93.33 (70.18–99.66)	73.33 (48.05–89.10)

a A total of 247 different combinations from seven categories were assessed; the 7 combinations with the best performance in each category are shown. TB, tuberculosis; LTBI, latent tuberculosis infection; AUC, areas under the receiver operator characteristic curve; CI, confidence interval; WHO TPP, World Health Organization target product profile.

### Functional analysis of difference methylated CpG sites.

**FIG 3:**
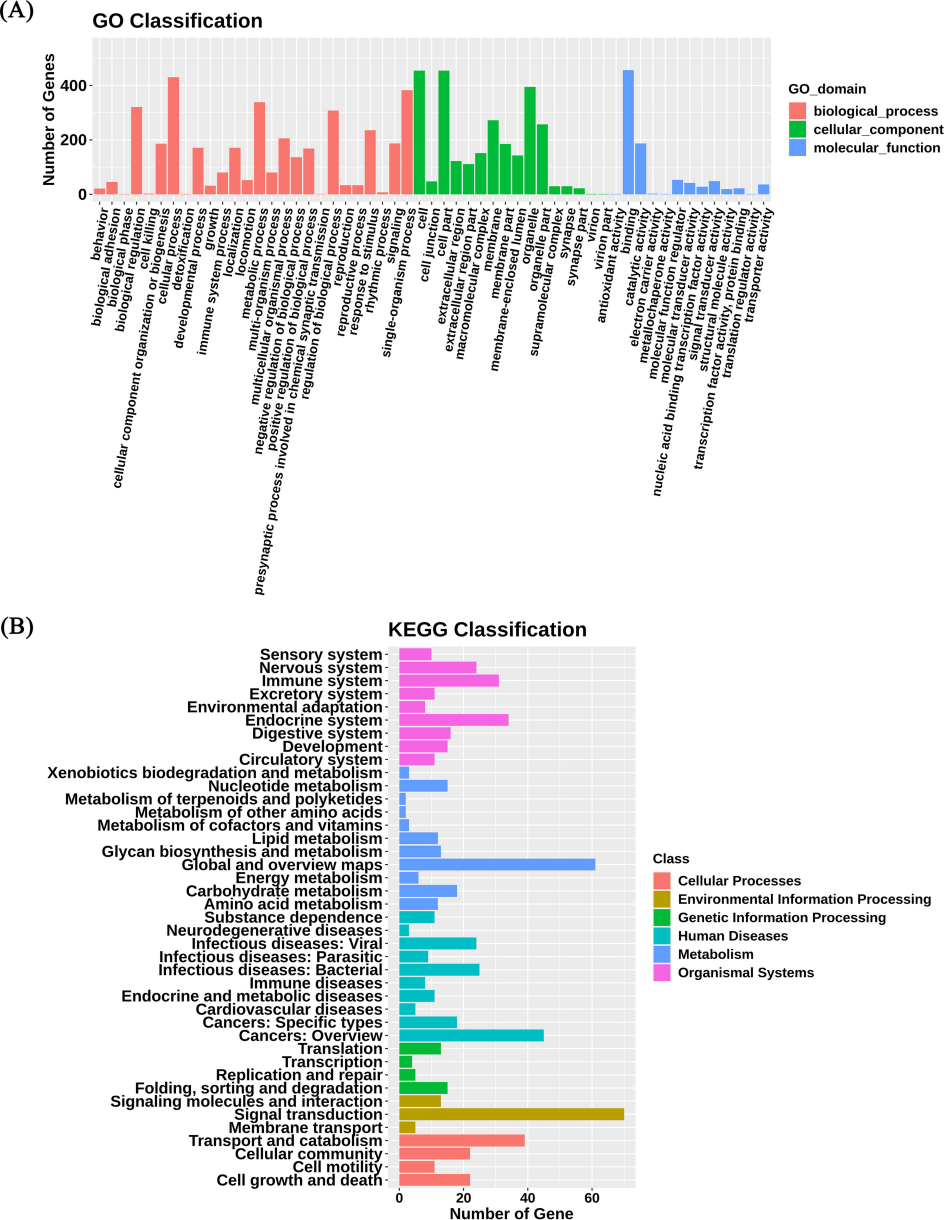
GO and KEGG classification. (A) GO classification map of differential methylation site-related genes. The abscissa represents GO classification, and the ordinate represents the number of genes, enriched GO classification on biological processes, cellular components, and molecular functions. (B) KEGG classification map of differential methylation site-related genes. The abscissa is the number of genes, the ordinate is the second classification of KEGG, and the same color indicates the first classification of KEGG. GO, gene ontology; KEGG, Kyoto Encyclopedia of Genes and Genomes.

To further analyze the biological function of the 2826 dmCpGs between case group and control group during follow-up, GO function and KEGG pathway enrichment analysis were conducted with DAVID. With GO function analysis, the results indicated that the dmCpGs were mostly enriched in the biological process of insulin secretion involved in cellular response to glucose stimulus, Fcγ receptor signaling pathway involved in phagocytosis, and regulation of the sequestering of calcium ion (Fig. 3A). The results of KEGG pathway analysis suggested, it was suggested that these dmCpGs were mainly enriched in bacterial infectious diseases and cancers (Fig. 3B).

## DISCUSSION

In this pilot study, eight dmCpGs (cg02493602, cg02206980, cg02214623, cg12159502, cg14593639, cg25764570, cg02781074, and cg12321798) were identified to be differently expressed between LTBI and active TB. The combination of “cg02206980 + cg02214623 + cg12159502 + cg12321798” showed the best performance, with a sensitivity of 93.33% and a specificity of 86.67%. The findings suggested that the varied DNA methylation profile at certain CpG cites might play a role in modulating host susceptibility to active TB occurrence and used as a potential biomarker to distinguish TB from LTBI.

The association between DNA methylation and TB risk was reported as early as 40 years ago (17). A wealth of studies reported aberrant DNA-methylated genes or global DNA methylation in TB patients (5, 18–22). A previous study using Illumina’s DNA methylation 450K assay identified differentially methylated loci between active pulmonary TB patients and healthy subjects. The study showed that varied DNA methylation over the PARP9/miR505/RASGRP4/GNG12 genes may regulate to the development of active TB onset (16). Another study evaluated the DNA methylation status of TB patients and their asymptomatic household contacts and found that patients with TB have DNA hypermethylation of the IL-2/STAT5, TNF/NF-κB, and IFN-γ signaling pathways, which demonstrated that TB patients are characterized by DNA hypermethylation of genes critical to mycobacterial immunity (15). However, most previous studies used healthy subjects as controls, which usually defined as asymptomatic individuals with normal chest X-ray examination results and had a negative history of TB disease. The infection status of such controls was unknown. As has been reported by a longitudinal study which found healthy subjects who later developed LTBI had a unique DNA methylation profile (23), the aberrant DNA-methylated genes detected in aforementioned studied might confounded by LTBI status of the controls because of the complex immune progress from TB infection to active disease. Esterhuyse et al. (24) incorporated dmCpGs from only monocytes and granulocytes by machine learning to distinguish active TB from LTBI in cross-sectional design and obtained a model with an AUC of 0.74, which was consistent with our results. To our knowledge, the present study is the first to identify distinct differential DNA methylation profiles between individuals with LTBI who later developed or did not develop TB. The longitudinal self-control design might help us to determine whether the varied host genomic methylation is due to host genetic polymorphisms or is caused by M. tuberculosis infection and pathogenesis. Exploring diagnostic biomarkers that could be of help in identifying active TB from LTBI is meaningful for the practice of precise intervention. Our understanding of these epigenetic changes will enable the use of epigenetic biomarkers for the diagnosis of disease in early stages.

Targeting populations with the high priority for preventive treatment was the first step for implementation of intervention. Thus, systematic screening for TB disease among high-risk groups could not only achieve early detection and treatment but also identify individuals who are eligible for and would benefit from TB preventive treatment once TB disease was ruled out. In consideration of the subclinical TB (25) and paucibacillary nature of culture-negative TB, blood-based, host-derived immune response biomarkers might improve diagnostic sensitivity compared to microbiologically based methods (26). In addition, TB screening tools are not intended to provide a definitive diagnosis. In a screening context, the most desirable strategy would be one with a high total yield of true-positive TB cases, has few false-positive results, is low in cost, and can be performed quickly, whereas many of these factors tend to counteract one another in clinical practice (4). Thus, in 2014, the WHO released a report highlighting that the minimal requirements for a target screening test would be an overall sensitivity of 90% and a specificity of 70% (27). According to these criteria, we found several eligible combinations of the eight dmCpGs that met the requirements, although this needs further verification.

Among the many combinations of the eight candidate dmCpGs, increasing the numbers of CpGs did not add extra diagnostic value. One combination of four CpGs—cg02206980 + cg02214623 + cg12159502 + cg12321798—showed the best performance, which indicated their located genes might involve in TB pathogenesis. cg12159502-located gene Sirt1 and cg02206980-located gene Sirt5 belong to the Sirtuins family, which is a class of NAD-dependent histone deacetylases that share various functions related to energy homeostasis, stress response, and tumorigenesis (28). It was reported that Sirt1 was required in the inhibition of apoptosis and inflammatory responses in human cells and had been frequently reported to be related to TB through inflammatory responses (29–31). The activated SIRT1 deacetylates MAP1LC3B/LC3B to induce its translocation into the cytoplasm and activate autophagy, which is this pathway’s physiological role in autophagy-mediated trafficking of M. tuberculosis into lysosomes to restrict intracellular mycobacteria growth (32). Another study also reported Sirt1 acted as a novel regulator of apoptosis signaling in M. tuberculosis infection via its direct effects on GSK3β (33). Therefore, the hypermethylation of Sirt1 in case group may participate in the process of active TB development by inhibiting the expression of Sirt1 mRNA and interfering the M. tuberculosis apoptosis. Diabetes mellitus is one risk factor for the development of active TB due to impaired production of chemokines and cytokines (34). A previous study demonstrated that SIRT5 has a potential role in regulating glucose homeostasis, and Sirt5 deficiency mice boost IL-1β production in inflammatory response (35). These findings provided us with new insight to further explore potential immune mechanisms of Sirt5 in TB development in diabetes patients. No study has reported the relation of the cg02214623-located gene GNB2L1 and the cg12321798-located gene FLJ44635 to TB. The underlying mechanisms need to be verified and explored further in future studies.

The present study faced a number of limitations. First, our study participants were middle aged and elderly rural residents. Therefore, our findings cannot be simply extended to other populations. In addition, the sample size of the present study was small, and only a few TB cases occurred during 5-year follow-up period; this meant subgroup analysis on the diagnostic times was unavailable. Thus, the identified dmCpGs in our pilot study need to be verified in further independent populations with large sample sizes. Second, to explore the most promising potential methylated CpG sites related to TB development, strict criteria were set in our study; thus, some relevant methylated CpG sites might be over-excluded. Third, a total of 134 dmCpGs were found and distributed among different regions according to our selection criteria; since only dmCpGs in the TSS200 or TSS1500 region (located in the promoter region) were selected for further analysis, we cannot exclude the potential value of dmCpGs located in other regulatory regions in distinguishing active TB from LTBI.

### Conclusion.

Our preliminary results indicate that varied DNA methylation levels might be related to alterations in the expression of certain genes during active TB occurrence, which might provide potential value as a diagnostic biomarker for discriminating active disease in the case of LTBI testing. Further studies are warranted to verify the findings in different study populations with large sample sizes.

## MATERIALS AND METHODS

### Study design and population.

The present nested case-control study was based on an open-label randomized controlled trial that aiming to explore short-course LTBI treatment regimens for rural residents aged 50 to 70 years in 2015. Detailed information on the study design has been published elsewhere (36, 37). At baseline, all eligible participants with QuantiFERON-TB Gold In-Tube (Qiagen, USA)-positive results (a cutoff value of ≥ 0.35 IU/mL was used, as recommended by the manufacturer) and without current active TB at baseline survey were included, and 3-mL blood samples were collected. Then, 3,783 study participants were randomized into three groups (two intervention groups and one untreated control group) and followed up for 5 years to track the development of active TB. TB cases were defined according to the National Guideline for the Diagnosis of Pulmonary Tuberculosis (WS 288–2017). For microbiologically confirmed or clinical diagnosis TB cases, 3-mL venous blood samples were collected before initiating antituberculosis treatment. In present study, in order to avoid the influence of preventative treatment on TB incidence, only 1,155 participants from the untreated group were included. During the 5-year follow-up, 26 individuals identified with active TB and 15 identified with sufficient peripheral blood mononuclear cells (PBMCs) were selected as the case group for DNA methylation analyses. Fifteen age- and gender-matched subjects who remained free of TB were randomly selected from the rest of the individuals with LTBI to serve as a control group. The study was conducted in accordance with the Declaration of Helsinki, and written informed consent was obtained from each participant.

### Illumina Infinium MethylationEPIC BeadChip array.

Two sets of 3-mL blood samples were collected from each subject and tested, one sample at baseline and one sample at diagnosis, for those developed active TB or at terminal survey for those stayed healthy during follow-up. PBMCs were isolated from platelet-depleted whole blood using standard Ficoll-Paque density gradient centrifugation, and genomic DNA from PBMCs extracted using a Puregene Core kit (Qiagen, Hilden, Germany). DNA (500 ng) was treated with bisulfate using an EZ DNA Methylation Gold kit (Zymo Research, Irvine, CA) according to the manufacturer’s instructions. The methylation of DNA was assayed on a Methylation 850K Beadchip (Illumina, San Diego, CA) using an Illumina HD methylation assay kit (Shanghai Biotechnology Corporation).

DNA methylation data were analyzed using the methylation analysis module within BeadStudio software employing default parameters (Illumina, Inc., San Diego, CA). The raw intensity data were loaded to a biocondutor package “minfi.” The raw data were normalized using the subset-quantile-within-array normalization method, and probes with a detection *P* value of ≥0.01 in at least one sample were excluded from further analysis. Methylation values, referred to as β values, were calculated as the ratio of the methylated signal intensity to the sum of the methylated and unmethylated signals after background subtraction, ranging from 0 (completely unmethylated) to 1 (fully methylated). Specifically, we considered a probe to be differentially methylated if the absolute Δβ was >0.1 and the statistical test was significant (*P* < 0.05).

### Criteria for differential DNA methylation analyses.

Differentially methylated CpG sites (dmCpGs) were selected using an algorithm in an IMA Bioconductor. Here, we assessed the mean-difference β-value (Δβ) between the two sample groups for each CpG site. In the present study, dmCpGs between the groups were identified with |Δβ| > 0.10 and *P < *0.05. Only dmCpGs that met following criteria were further selected as candidate CpG sites. First, the dmCpGs existed between the case group and the control group in follow-up samples. Second, the dmCpGs should only be found between baseline samples and follow-up samples in the case group rather than in the control group. Third, only dmCpGs located in the promoter regions (promoters were defined as regions located between 1,500 bp upstream of transcriptional start sites [TSS] and 200 bp downstream of TSS) were selected.

### Functional annotations.

Gene ontology (GO) analysis of the methylation profile was performed using the “clusterProfiler” package. We used the Benjamini-Hochberg method to determine the adjusted *P* values, and pathways in which false discovery rate values were <0.05 were chosen. The Kyoto Encyclopedia of Genes and Genomes (KEGG) was used to identify the exact enriched genes for specific biological terms or pathways.

### Statistical analyses.

The Statistical Analysis System (SAS 9.4 for Windows; SAS Institute, Inc., Cary, NC) was used for data analyses. Chi-square and Fisher exact tests were used to compare the distribution of categorical variables. The numerical variables are presented as median and Q25–Q75 (25th to 75th percentile) values. Wilcoxon rank sum tests were used to compare continuous variable. Volcano plots and cluster analysis were conducted to present the dmCpGs. ROC curve and AUC analyses were performed to evaluate the diagnostic ability of the dmCpGs for discriminating TB disease from LTBI. Sensitivities and specificities were calculated using the highest Youden index value as the cutoff. We also compared the performance of individual methylated CpG sites or of combined methylated CpGs according to WHO’s TPP for a diagnostic tool of at least 90% sensitivity by altering the threshold to match each target value. *P* < 0.05 was considered statistically significant.

### Data availability.

All data generated or analyzed during this study are included in this published article. Raw data can be uploaded upon request.
