# Supplementary material for: Relationship between DNA Methylation Profiles and Active Tuberculosis Development from Latent Infection: a Pilot Study in Nested Case-Control Design
Source: Microbiol Spectr. 2022 Apr 21;10(3):e00586-22. doi: 10.1128/spectrum.00586-22 (PMC9241819; doi:10.1128/spectrum.00586-22)
Supplement: SUPPLEMENTAL FILE 1 — Tables S1 and s2. Download spectrum.00586-22-s001.pdf, PDF file, 0.3 MB [file spectrum.00586-22-s001.pdf]

Supporting Information

The relationship between DNA methylation profiles and active tuberculosis development from latent infection: a pilot study in nested case-control design

Ying Du [1], Xu Gao [2], Jiaoxia Yan [3], Haoran Zhang [1], Xuefang Cao [1], Boxuan Feng [1], Yijun He [1], Yongpeng He [1], Tonglei Guo [1], Henan Xin[1]*, Lei Gao[1]*

[1]NHC Key Laboratory of Systems Biology of Pathogens, Institute of Pathogen Biology, and Center for Tuberculosis Research, Chinese Academy of Medical Sciences and Peking Union Medical College, Beijing 100730, P.R. China.

[2]Department of Occupational and Environmental Health Sciences, School of Public Health, Peking University, Beijing 100190, China.

[3]Center for Diseases Control and Prevention of Zhongmu, Zhengzhou 451450, China.

*** Correspondence:**

Prof. Lei Gao and Prof. Henan Xin, NHC Key Laboratory of Systems Biology of Pathogens, Institute of Pathogen Biology, Chinese Academy of Medical Sciences and Peking Union Medical College, No 9 Dong Dan San Tiao, Beijing 100730, China. Email: gaolei@ipbcams.ac.cn, xinhenan@ipbcams.ac.cn.

Supplementary Table 1 Detailed information of the active tuberculosis cases identified during 5-year follow-up

ID	Time for baseline survey	Time for diagnosis	Smear	Culture	Gene X-pert	Diagnosis
1042040381	Jul-2015	Aug-2020	negative	positive	positive	Microbiological confirmed
3152060882	Jul-2015	Aug-2020	negative	positive	negative	Microbiological confirmed
4141040201	Jul-2015	Aug-2020	negative	positive	positive	Microbiological confirmed
5041030051	Jul-2015	Aug-2020	negative	positive	positive	Microbiological confirmed
6066020021	Jul-2015	Aug-2020	positive	positive	positive	Microbiological confirmed
1121010061	Jul-2015	Aug-2020	negative	positive	positive	Microbiological diagnosis
3073100012	Jul-2015	Aug-2020	positive	positive	positive	Microbiological diagnosis
3111010092	Jul-2015	Aug-2020	negative	positive	positive	Microbiological diagnosis
3141020351	Jul-2015	Aug-2020	negative	positive	positive	Microbiological diagnosis
3431010061	Jul-2015	Aug-2020	negative	positive	positive	Microbiological diagnosis
5071050032	Jul-2015	Aug-2020	negative	positive	negative	Microbiological diagnosis
1181040172	Jul-2015	Dec-2019	negative	negative	negative	Clinical diagnosis
3063040171	Jul-2015	Dec-2019	negative	negative	negative	Clinical diagnosis
7031040361	Jul-2015	Dec-2019	negative	negative	negative	Clinical diagnosis
1032040081	Jul-2015	Nov-2017	negative	negative	negative	Clinical diagnosis

Supplementary Table 2 The performance of 52 combinations met the WHO TPP benchmarks for screening test (>90% sensitivity and >70% specificity).

Combined	AUC	*P* value	Maximum Youden index		WHO TPP benchmarks	
			Sensitivity,%	Specificity,%	Sensitivity,%	Specificity,%
c2c3c4	0.89 (0.72, 0.97)	<0.001	93.33 (70.18, 99.66)	80.00 (54.81, 92.95)	93.33 (70.18, 99.66)	80.00 (54.81, 92.95)
c2c4c7	0.84 (0.66, 0.95)	<0.001	93.33 (70.18, 99.66)	73.33 (48.05, 89.10)	93.33 (70.18, 99.66)	73.33 (48.05, 89.10)
c4c5c7	0.86 (0.68, 0.96)	<0.001	93.33 (70.18, 99.66)	73.33 (48.05, 89.10)	93.33 (70.18, 99.66)	73.33 (48.05, 89.10)
c4c7c8	0.85 (0.68, 0.96)	<0.001	93.33 (70.18, 99.66)	73.33 (48.05, 89.10)	93.33 (70.18, 99.66)	73.33 (48.05, 89.10)
c1c2c3c4	0.88 (0.72, 0.97)	<0.001	86.67 (62.12, 97.63)	80.00 (54.81, 92.95)	93.33 (70.18, 99.66)	73.33 (48.05, 89.10)
c1c4c5c7	0.86 (0.68, 0.96)	<0.001	93.33 (70.18, 99.66)	73.33 (48.05, 89.10)	93.33 (70.18, 99.66)	73.33 (48.05, 89.10)
c2c3c4c5	0.88 (0.72, 0.97)	<0.001	93.33 (70.18, 99.66)	80.00 (54.81, 92.95)	93.33 (70.18, 99.66)	80.00 (54.81, 92.95)
c2c3c4c6	0.88 (0.72, 0.97)	<0.001	86.67 (62.12, 97.63)	80.00 (54.81, 92.95)	93.33 (70.18, 99.66)	73.33 (48.05, 89.10)
c2c3c4c7	0.88 (0.72, 0.97)	<0.001	86.67 (62.12, 97.63)	86.67 (62.12, 97.63)	93.33 (70.18, 99.66)	73.33 (48.05, 89.10)
c2c3c4c8	0.88 (0.72, 0.97)	<0.001	93.33 (70.18, 99.66)	86.67 (62.12, 97.63)	93.33 (70.18, 99.66)	86.67 (62.12, 97.63)
c2c4c5c7	0.84 (0.66, 0.95)	<0.001	93.33 (70.18, 99.66)	73.33 (48.05, 89.10)	93.33 (70.18, 99.66)	73.33 (48.05, 89.10)

c2c4c7c8	0.85 (0.68, 0.96)	<0.001	93.33 (70.18, 99.66)	73.33 (48.05, 89.10)	93.33 (70.18, 99.66)	73.33 (48.05, 89.10)
c3c4c5c8	0.91 (0.75, 0.98)	<0.001	86.67 (62.12, 97.63)	86.67 (62.12, 97.63)	93.33 (70.18, 99.66)	73.33 (48.05, 89.10)
c4c5c6c7	0.87 (0.69, 0.96)	<0.001	93.33 (70.18, 99.66)	73.33 (48.05, 89.10)	93.33 (70.18, 99.66)	73.33 (48.05, 89.10)
c4c5c6c8	0.87 (0.69, 0.96)	<0.001	100.00 (93.47, 100.00)	73.33 (48.05, 89.10)	93.33 (70.18, 99.66)	73.33 (48.05, 89.10)
c4c5c7c8	0.86 (0.68, 0.96)	<0.001	93.33 (70.18, 99.66)	73.33 (48.05, 89.10)	93.33 (70.18, 99.66)	73.33 (48.05, 89.10)
c1c2c3c4c5	0.88 (0.72, 0.97)	<0.001	93.33 (70.18, 99.66)	80.00 (54.81, 92.95)	93.33 (70.18, 99.66)	80.00 (54.81, 92.95)
c1c2c3c4c7	0.88 (0.72, 0.97)	<0.001	86.67 (62.12, 97.63)	80.00 (54.81, 92.95)	93.33 (70.18, 99.66)	73.33 (48.05, 89.10)
c1c2c3c4c8	0.89 (0.72, 0.97)	<0.001	93.33 (70.18, 99.66)	80.00 (54.81, 92.95)	93.33 (70.18, 99.66)	80.00 (54.81, 92.95)
c1c2c4c5c7	0.84 (0.66, 0.95)	<0.001	93.33 (70.18, 99.66)	73.33 (48.05, 89.10)	93.33 (70.18, 99.66)	73.33 (48.05, 89.10)
c1c4c5c6c7	0.86 (0.68, 0.96)	<0.001	93.33 (70.18, 99.66)	73.33 (48.05, 89.10)	93.33 (70.18, 99.66)	73.33 (48.05, 89.10)
c1c4c5c6c8	0.88 (0.72, 0.97)	<0.001	80.00 (54.81, 92.95)	86.67 (62.12, 97.63)	93.33 (70.18, 99.66)	73.33 (48.05, 89.10)
c2c3c4c5c6	0.88 (0.72, 0.97)	<0.001	93.33 (70.18, 99.66)	80.00 (54.81, 92.95)	93.33 (70.18, 99.66)	80.00 (54.81, 92.95)
c2c3c4c5c7	0.88 (0.72, 0.97)	<0.001	86.67 (62.12, 97.63)	86.67 (62.12, 97.63)	93.33 (70.18, 99.66)	73.33 (48.05, 89.10)
c2c3c4c5c8	0.89 (0.72, 0.97)	<0.001	93.33 (70.18, 99.66)	86.67 (62.12, 97.63)	93.33 (70.18, 99.66)	86.67 (62.12, 97.63)

c2c3c4c6c8	0.89 (0.72, 0.97)	<0.001	93.33 (70.18, 99.66)	86.67 (62.12, 97.63)	93.33 (70.18, 99.66)	86.67 (62.12, 97.63)
c2c3c4c7c8	0.89 (0.72, 0.97)	<0.001	93.33 (70.18, 99.66)	80.00 (54.81, 92.95)	93.33 (70.18, 99.66)	80.00 (54.81, 92.95)
c2c4c5c6c7	0.86 (0.68, 0.96)	<0.001	93.33 (70.18, 99.66)	73.33 (48.05, 89.10)	93.33 (70.18, 99.66)	73.33 (48.05, 89.10)
c2c4c5c6c8	0.87 (0.69, 0.96)	<0.001	100.00 (93.47, 100.00)	73.33 (48.05, 89.10)	93.33 (70.18, 99.66)	73.33 (48.05, 89.10)
c2c4c5c7c8	0.86 (0.68, 0.96)	<0.001	93.33 (70.18, 99.66)	73.33 (48.05, 89.10)	93.33 (70.18, 99.66)	73.33 (48.05, 89.10)
c3c4c5c6c8	0.91 (0.75, 0.98)	<0.001	86.67 (62.12, 97.63)	86.67 (62.12, 97.63)	93.33 (70.18, 99.66)	73.33 (48.05, 89.10)
c3c4c5c7c8	0.91 (0.75, 0.98)	<0.001	86.67 (62.12, 97.63)	80.00 (54.81, 92.95)	93.33 (70.18, 99.66)	73.33 (48.05, 89.10)
c1c2c3c4c5c6	0.88 (0.72, 0.97)	<0.001	93.33 (70.18, 99.66)	80.00 (54.81, 92.95)	93.33 (70.18, 99.66)	80.00 (54.81, 92.95)
c1c2c3c4c5c7	0.88 (0.72, 0.97)	<0.001	86.67 (62.12, 97.63)	80.00 (54.81, 92.95)	93.33 (70.18, 99.66)	73.33 (48.05, 89.10)
c1c2c3c4c5c8	0.90 (0.73, 0.98)	<0.001	93.33 (70.18, 99.66)	80.00 (54.81, 92.95)	93.33 (70.18, 99.66)	80.00 (54.81, 92.95)
c1c2c3c4c6c8	0.90 (0.73, 0.98)	<0.001	93.33 (70.18, 99.66)	80.00 (54.81, 92.95)	93.33 (70.18, 99.66)	80.00 (54.81, 92.95)
c1c2c3c4c7c8	0.89 (0.72, 0.97)	<0.001	93.33 (70.18, 99.66)	80.00 (54.81, 92.95)	93.33 (70.18, 99.66)	80.00 (54.81, 92.95)
c1c2c4c5c6c7	0.86 (0.68, 0.96)	<0.001	93.33 (70.18, 99.66)	73.33 (48.05, 89.10)	93.33 (70.18, 99.66)	73.33 (48.05, 89.10)
c1c2c4c5c6c8	0.87 (0.69, 0.96)	<0.001	80.00 (54.81, 92.95)	86.67 (62.12, 97.63)	93.33 (70.18, 99.66)	73.33 (48.05, 89.10)

c1c2c4c5c7c8	0.86 (0.68, 0.96)	<0.001	93.33 (70.18, 99.66)	73.33 (48.05, 89.10)	93.33 (70.18, 99.66)	73.33 (48.05, 89.10)
c1c3c4c5c7c8	0.91 (0.75, 0.98)	<0.001	80.00 (54.81, 92.95)	86.67 (62.12, 97.63)	93.33 (70.18, 99.66)	73.33 (48.05, 89.10)
c2c3c4c5c6c7	0.88 (0.72, 0.97)	<0.001	86.67 (62.12, 97.63)	86.67 (62.12, 97.63)	93.33 (70.18, 99.66)	73.33 (48.05, 89.10)
c2c3c4c5c6c8	0.89 (0.72, 0.97)	<0.001	93.33 (70.18, 99.66)	86.67 (62.12, 97.63)	93.33 (70.18, 99.66)	86.67 (62.12, 97.63)
c2c3c4c5c7c8	0.89 (0.72, 0.97)	<0.001	93.33 (70.18, 99.66)	80.00 (54.81, 92.95)	93.33 (70.18, 99.66)	80.00 (54.81, 92.95)
c2c3c4c6c7c8	0.88 (0.72, 0.97)	<0.001	93.33 (70.18, 99.66)	80.00 (54.81, 92.95)	93.33 (70.18, 99.66)	80.00 (54.81, 92.95)
c3c4c5c6c7c8	0.91 (0.75, 0.98)	<0.001	86.67 (62.12, 97.63)	80.00 (54.81, 92.95)	93.33 (70.18, 99.66)	73.33 (48.05, 89.10)
c1c2c3c4c5c6c7	0.90 (0.73, 0.98)	<0.001	86.67 (62.12, 97.63)	80.00 (54.81, 92.95)	93.33 (70.18, 99.66)	73.33 (48.05, 89.10)
c1c2c3c4c5c6c8	0.87 (0.69, 0.96)	<0.001	93.33 (70.18, 99.66)	80.00 (54.81, 92.95)	93.33 (70.18, 99.66)	80.00 (54.81, 92.95)
c1c2c3c4c5c7c8	0.90 (0.73, 0.98)	<0.001	86.67 (62.12, 97.63)	86.67 (62.12, 97.63)	93.33 (70.18, 99.66)	73.33 (48.05, 89.10)
c1c2c3c4c6c7c8	0.89 (0.72, 0.97)	<0.001	93.33 (70.18, 99.66)	80.00 (54.81, 92.95)	93.33 (70.18, 99.66)	80.00 (54.81, 92.95)
c2c3c4c5c6c7c8	0.90 (0.73, 0.98)	<0.001	93.33 (70.18, 99.66)	80.00 (54.81, 92.95)	93.33 (70.18, 99.66)	80.00 (54.81, 92.95)
c1c2c3c4c5c6c7c8	0.90 (0.73, 0.98)	<0.001	86.67 (62.12, 97.63)	86.67 (62.12, 97.63)	93.33 (70.18, 99.66)	73.33 (48.05, 89.10)

Abbreviation: WHO TPP, World health organization target product profile; c1, cg02493602; c2, cg02206980; c3, cg02214623; c4, cg12159502; c5, cg14593639; c6, cg25764570; c7, cg02781074; c8, cg12321798; AUC, Areas under the Receiver operator characteristic curve; WHO TPP, World health organization target product profile.
